# Functional MRI study of feedback-based reinforcement learning in depression

**DOI:** 10.3389/fninf.2022.1028121

**Published:** 2022-12-20

**Authors:** Almira M. Kustubayeva, Erik B. Nelson, Michael L. Smith, Jane B. Allendorfer, James C. Eliassen

**Affiliations:** ^1^Center for Cognitive Neuroscience, Department of Physiology, Biophysics, and Neuroscience, Al-Farabi Kazakh National University, Almaty, Kazakhstan; ^2^Department of Psychiatry and Behavioral Neuroscience, College of Medicine, University of Cincinnati, Cincinnati, OH, United States; ^3^Department of Speech-Language-Hearing Sciences, University of Minnesota, Minneapolis, MN, United States; ^4^Department of Neurology, University of Alabama at Birmingham, Birmingham, AL, United States; ^5^Robert Bosch Automotive Steering LLC, Florence, KY, United States

**Keywords:** associative learning, reinforcement, functional magnetic resonance imaging (fMRI), major depressive disorder, brain activity

## Abstract

Reinforcement learning depends upon the integrity of emotional circuitry to establish associations between environmental cues, decisions, and positive or negative outcomes in order to guide behavior through experience. The emotional dysregulation characteristic of major depressive disorder (MDD) may alter activity in frontal and limbic structures that are key to learning. Although reward and decision-making have been examined in MDD, the effects of depression on associative learning is less well studied. We investigated whether depressive symptoms would be related to abnormalities in learning-related brain activity as measured by functional magnetic resonance imaging (fMRI). Also, we explored whether melancholic and atypical features were associated with altered brain activity. We conducted MRI scans on a 4T Varian MRI system in 10 individuals with MDD and 10 healthy subjects. We examined event-related brain activation during feedback-based learning task using Analysis of Functional NeuroImages (AFNI) for image processing and statistical analysis. We observed that MDD patients exhibited reduced activation in visual cortex but increased activation in cingulate and insular regions compared to healthy participants. Also, in relation to features of depressive subtypes, we observed that levels of activation in striatal, thalamic, and precuneus regions were negatively correlated with atypical characteristics. These results suggest that the effects of MDD change the neural circuitry underlying associative learning, and these effects may depend upon subtype features of MDD.

## Introduction

Major Depression Disorder is characterized by persistent, dysphoric emotions along with disturbances in motivated and psychomotor behavior, all of which interfere with activities of daily living. Depressive symptoms often accompany learning disabilities, but the evidence for the mechanisms of impaired learning in major depressive disorder (MDD) remains less certain (Hans, 1997; Bender et al., 1999). Learning yields behavioral adaptation to environmental changes by enabling individuals to associate positive and negative outcomes with prior behaviors. Learning disabilities may result from executive function deficits or emotion and reward processing abnormalities (Snyder, 2013; Knight and Baune, 2018; Wang et al., 2021). Empirical studies have reported impaired cognitive performance in MDD with attention and memory tasks related to hippocampal function (Weingartner et al., 1981; Dolan et al., 1992; Austin et al., 1999; Lin et al., 2014). Neuroimaging studies have identified brain dysfunction in patients with MDD during a variety of cognitive processes (Johnstone et al., 2007; Broyd et al., 2009; Smoski et al., 2009; Clark and Beck, 2010; Linden et al., 2012). Executive function deficits associated with altered prefrontal function and anterior cingulate cortex (ACC) activation have been described in several studies (Elliott et al., 1997, 1998; Austin et al., 2001). Impairments in emotion regulation related to prefrontal-subcortical abnormalities also contribute to depression vulnerability (Beauregard et al., 2006; Johnstone et al., 2007; Linden et al., 2012). A growing number of studies focusing on MDD symptom of anhedonia have demonstrated abnormalities in reward processing (Smoski et al., 2009, 2011; Zhang et al., 2013). The findings of altered reward processing are somewhat inconsistent. Hyposensitivity of striatal regions to reward feedback and reduced activation in the middle frontal gyrus and ACC have been reported in several studies (Schaefer et al., 2006; Forbes et al., 2009; Smoski et al., 2009). A meta-analysis of functional magnetic resonance imaging (fMRI) studies of reward processing in MDD supports the hypothesis of decreased subcortical and limbic activity in MDD patients (Zhang et al., 2013). However, other studies have reported hypersensitivity in the anterior insula to punishment and in the putamen to reward (Kumari et al., 2003; Remijnse et al., 2009), or no changes in reward areas during gain anticipation (Knutson et al., 2008). In these studies, the direction of effects to reward may depend on several factors including illness severity, medication history, and experimental task parameters. Impaired reward processing may underlie learning problems and behavioral adaptation (Chen et al., 2015). Reinforcement learning based on feedback is a more complex task which integrates sensory perception, reward processing, and motor action. Consequently, trial-and-error reinforcement learning may activate the different brain circuitry in comparison to simple reward processing. For instance, Gerraty et al. (2018) describe dynamical changes in brain networks and coupling between the striatum, visual, orbitofrontal, and ventromedial prefrontal cortex related to learning rate. Moreover, studies of reinforcement learning have been extended to computational models for outcome prediction (Daw et al., 2011) and its application to diagnostic and treatment methods (Brown et al., 2021; Heo et al., 2021). Therefore, it is important to understand the mechanism of reinforcement learning and how depressive symptoms such as emotion dysregulation and reward sensitivity affect the brain activation during learning. Also, positive and negative feedback itself may induce an emotional state corresponding to the valence of feedback (Kustubayeva et al., 2012). Previous learning research shows that healthy subjects exhibit prominent activation to the first trial feedback that diminishes on the next trial in a one-trial learning task (Eliassen et al., 2012). Feedback guides future behavior and the brain response to novel early feedback subsides dramatically as outcomes become predictable. It is important to learn how the integration of feedback and stimulus perception is changed in patients with MDD.

Our study evaluated the brain response to feedback-based visual-motor associative learning in healthy participants and MDD patients. We hypothesized that in comparison to healthy subjects MDD patients would show limbic-cortical dysregulation. We predicted that the presence of depression would lead to reduced activation during learning, suggesting less efficient information processing in MDD patients. Additionally, evidence from a number of studies suggests that depression is a heterogeneous disorder, with variations in phenomenology reflecting underlying neuropathophysiological differences (Fountoulakis et al., 2004; Baumeister and Parker, 2011; Foti et al., 2014; Day et al., 2015). This study recruited MDD patients who met criteria for a major depressive episode with either melancholic or atypical characteristics. Some studies show that atypical depression is distinguished from melancholia by increased right hemispheric processing and the right frontal perfusion (Fountoulakis et al., 2004). Because these subtypes differ with regard to neurovegetative symptoms and emotional reactivity, and sensitivity to reward, we also hypothesized that an altered response to learning would be associated with subtype characteristics. We present preliminary findings on these effects as well.

## Materials and methods

### Subjects

Twelve patients with MDD who met criteria for either atypical or melancholic subtypes of MDD (Mean Age = 40.5; SD = 7.92; 5 males, 7 females) and 10 healthy control subjects (Mean Age = 30.2; SD = 8.34; 5 males, 5 females) were recruited for this study *via* advertisement from outpatient and community populations. Inclusion criteria for both groups included age from 18 to 55, fluent in English and able to understand and provide written informed consent. MDD participants had to meet the following criteria: score on the Inventory of Depressive Symptomatology (IDS) scale higher than 20. Diagnosis of major depression was determined using the Structured Clinical Interview for DSM-IV (SCID), American Psychiatric Association [APA] (2000) and the presence of melancholic or atypical subtype was determined using the SCID supplements for these categories (Spitzer et al., 1990; First et al., 2002). Melancholic and atypical symptom score was based on sum of corresponding IDS items. Patients were unmedicated at the time of study participation and were excluded for use of any psychotropic medication in the past week, antidepressant medication, including herbal or natural substances purported to have antidepressant properties (e.g., St. John’s Wort, SAM-e, etc.) within the past 2 weeks, or the use of fluoxetine within the past 4 weeks. Two patients data were excluded due to the fMRI artifacts. To avoid the potential confounding effects of medication on blood oxygen level dependent (BOLD) signal changes, patients were included only if they had taken no antidepressant medications within 2 weeks of the scanning session. Also, participants were excluded if they had an unstable medical condition or and substance use disorder with the past 3 months.

Healthy subjects were included based on: (1) no history of DSM-IV Axis I disorder by history or by SCID interview; (2) no first-degree relative with a known history of a mood, anxiety, or substance use disorder. The study was approved by the Institutional Review Board (IRB) of the University of Cincinnati, and all subjects participated in the experiment after first providing written informed consent. Demographic, clinical and education information are summarized in Table 1. Education level was estimated by using the categories: some high school (0); graduated high school or Graduate Equivalency Degree (GED) (1); part college (2); completed 2 years of college (3); graduated 4 year college (4) and; graduate/professional school (5).

**TABLE 1:** Demographic and symptom severity of the participants.

	Depressed subjects mean (SD)	Control subjects mean (SD)	Differences between groups
*N*	10	10	–
Female: Male	5:5	5:5	–
Age	41.6 (6.47)	30.2 (8.34)	*F* = 8.79, *p* = 0.008
***Ethnicity***			
African American	20%	20%	–
Caucasian	70%	60%	–
Asian	10%	10%	–
Hispanic ethnicity		10%	–
Education level	3.09 (1.27)	3.14 (1.44)	*F* = 0.005 *p* = 0.94
Years since first depressive episode	13.2 (9.08)	n/a	n/a
Number of depressive episodes	3.2 (3.22)	n/a	n/a
Inventory of depressive symptomatology	37.8 (5.82)	0.80 (1.48)	*F* = 378.87 *P* < 0.001
Melancholic symptom score^a^	12.7 (5.06)	n/a	n/a
Atypical symptom score^b^	5.6 (4.86)	n/a	n/a

^a^Score based on sum of IDS items 4, 8, 12, 14, 29, and 30.

^b^Score based on sum of IDS items 3, 8, 9, 10, 11, 13, 21, 23, 24, and 31.

### Behavioral testing

#### Neuroimaging paradigm

Following clinical and behavioral testing, patients participated in a functional neuroimaging session at the University of Cincinnati’s Center for Imaging Research (CIR). After arriving at the CIR each participant was given oral and written instructions and performed a brief practice of the task with pictures different from those used during scanning. Subjects were provided with video goggles to view the computer screen during scanning, and a button box held in the right hand was used to record participants’ responses to the task. MRI-compatible headphones and a microphone enabled communication with the patient during the scan.

#### Functional magnetic resonance imaging behavioral task

**FIGURE 1:**
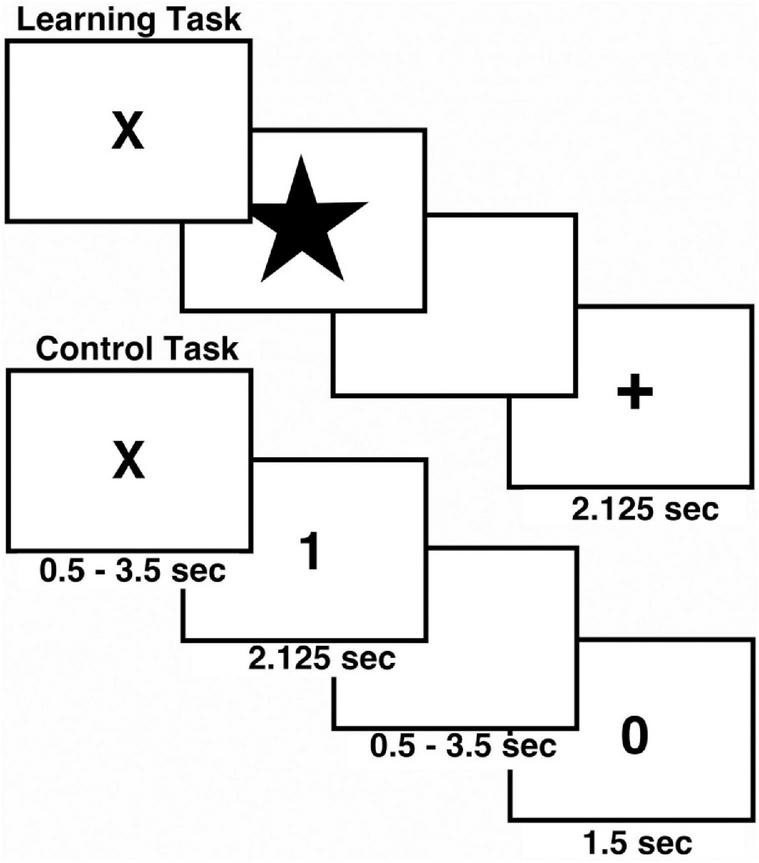
Schematic view of Associative Learning Task. The task included both control (*N* = 16) and learning trials (*N* = 32) in each run. The task was repeated twice during MRI scanning.

The associative learning task included both control and associative learning trials and was programmed in E-Prime^1^ to allow acquisition of behavioral performance (Figure 1; Eliassen et al., 2003, 2012). Two runs of the task were conducted during fMRI scanning. The control task consisted of 16 trials, and the learning task included 32 trials. During control trials the digit “1” or “2” was presented on the screen and participants pressed button 1 or 2 on the response box. During the learning trials, participants viewed easily named color pictures and learned to associate each picture with button 1 or 2 by trial-and-error using feedback (Rossion and Pourtois, 2004). A different set of four pictures was used for each run. Each picture was presented eight times pseudorandomized according to Latin Squares. A trial began with the presentation of a fixation cross (“X”). Following a brief variable delay a picture or digit appeared on screen, and subjects responded. After 2 s the screen went blank for a brief variable delay followed by feedback. Participants were instructed to make their responses during stimulus presentation. Positive feedback was indicated by a “+,” errors were indicated by “0,” and a lack of response resulted in a “?” Unlike our previous work with associative learning (Eliassen et al., 2012), the current task did not involve monetary rewards. For statistical analysis of the fMRI data we characterized learning by the number of correct responses to a picture. Learning or “experience” was divided into three levels, control, early learning (including the 1st trials, and the 2nd and 3rd correct trials) and late learning (all correct trials from 4th to 8th). Experience was then used as a factor in statistical analyses.

### Functional magnetic resonance imaging experiment

#### Image acquisition

Participants were scanned in a 4T Varian INOVA MRI system. A high-resolution T1-weighted 3-dimensional anatomical image was acquired using a Modified Driven Equilibrium Fourier Transform (MDEFT) acquisition^22^ (*T*_MD_ = 1.1 s, TR/TE = 13/6 ms, FOV = 256 × 192 × 192 mm, matrix 256 × 192 × 96 mm, voxel resolution 1 × 1 × 2 mm^3^, flip angle = 20°), zero-filled to 1 × 1 × 1 mm during reconstruction (Lee et al., 1995). For fMRI measurements we acquired two runs of T2*-weighted gradient-echo echoplanar images (EPI) consisting of 35 contiguous 5 mm-thick coronal slices covering the entire brain (TR/TE = 3,000/29 ms, FOV = 208 × 208 mm, matrix 64 × 64, flip angle = 75°, voxel resolution 3.25 × 3.25 × 5 mm) tuned to BOLD contrast and a multi-echo reference scan to correct geometric distortion and Nyquist ghost (Kwong et al., 1992; Ogawa et al., 1992; Schmithorst et al., 2001). Two 2-min runs were administered, and 146 volumes were acquired in each run. Two volumes at the beginning of each run were discarded to account for magnetization equilibrium effects.

#### MRI analysis

Functional images were reconstructed using in-house software written in Interactive Data Language (IDL)^2^ and included a 2D Hamming filter in the XY plane, which smoothed the resulting images. All further image processing including co-registration, motion correction, normalization, re-sampling, and event-related analysis were conducted in Analysis of Functional NeuroImages (AFNI) software (Cox, 1996; Cox and Hyde, 1997; Cox and Jesmanowicz, 1999). Fourier interpolation using a six-parameter rigid-body transformation was used for motion correction (Cox and Jesmanowicz, 1999). Each subject’s MDEFT image was normalized to Talairach space by alignment to the international consortium for brain mapping (ICBM) 452 brain template in AFNI.^3^ A 6 mm Gaussian smoothing was applied to the EPI data. EPI data sets were then normalized by adopting the MDEFT transform and resampled to 3 × 3 × 3 mm. Smoothing plus Hamming filtering resulted in 9 mm blurring in the XY (coronal) plane and 6 mm in the *Z* plane. The Monte Carlo simulations used to estimate statistical significance took this smoothing into account. Two subjects were excluded due to shim power supply hardware problems.

In order to estimate brain activation, a reference waveform was created for each subject by convolving specific task event times with a canonical hemodynamic response function (HRF) in AFNI. The canonical function incorporated a delay time of 2 s, a rise time of 4 s, and a fall time 4 s as we have used previously (Eliassen et al., 2012) and based on the timing of observed hemodynamic responses in our previous work (Eliassen et al., 2003). Signal drift was accounted for in each run by including polynomial regressors up to the third order (average, linear, quadratic, and cubic) and six motion correction parameters as regressors of no interest. Using AFNI’s 3dREMLfit, which accounts for serial auto correlations in the fMRI time series, we calculated fit coefficients to the polynomial regressors, the motion correction parameters and the reference waveforms representing the task events, stimulus, and feedback presentation for early learning, late learning, and control trials.

### Statistical analyses

Behavioral analyses were conducted using a mixed effects analysis approach in Statistical Analysis Software (SAS Institute Inc., SAS 9.1.3 Help and Documentation, Cary, NC, United States: SAS Institute Inc., 2002–2004). Reaction time data was calculated by evaluating the change in response times across all levels of experience [Early: 1st trials (correct or incorrect), Correct 2nd, and Correct 3rd trials; Late: Correct 4th–8th trials; and Control Trials].

Analysis of Functional NeuroImages’ 3dMVM program, utilizing R for statistical computing, was used to examine the group fMRI data, accounting for repeated measures and subjects as a random factor. For fMRI the dependent measures were the fit coefficient maps representing control, early, and late learning for both stimulus and feedback events. Several analyses were conducted with the fMRI data. Two analyses compared depression patients to healthy comparison subjects: one examined stimulus processing and the other examined feedback. Factors in the analyses included group (patients vs. healthy), experience (early vs. late learning trials), and event types (stimulus vs. feedback). We included an interaction term for group by experience. We examined the correlation between atypical score and stimulus or feedback activation for learning trials. Because age was significantly lower in the healthy group (*p* < 0.01) we included age as a covariate in the statistical examination of behavioral and fMRI data. Functional Regions-of-interest (ROI) were obtained from the activation maps and average signal intensity was extracted for each individual for different task events. Significant thresholds were determined for all brain imaging comparisons according to the Monte Carlo simulation tools used in AFNI (Friston et al., 1993; Forman et al., 1995; Xiong et al., 1995). A significant cluster was defined as a corrected *p*-value of 0.01, using a voxel-level *p*-value ≤0.005 and a minimum cluster size of 37 voxels. Talairach daemon (Lancaster et al., 2000) was used in order to identify the locations of activation clusters as well as the Montreal Neurological Institutes (Tzourio-Mazoyer et al., 2002) automated anatomical labels of single-subject high resolution T1 volumes as implemented in AFNI.

## Results

### Behavior

**FIGURE 2:**
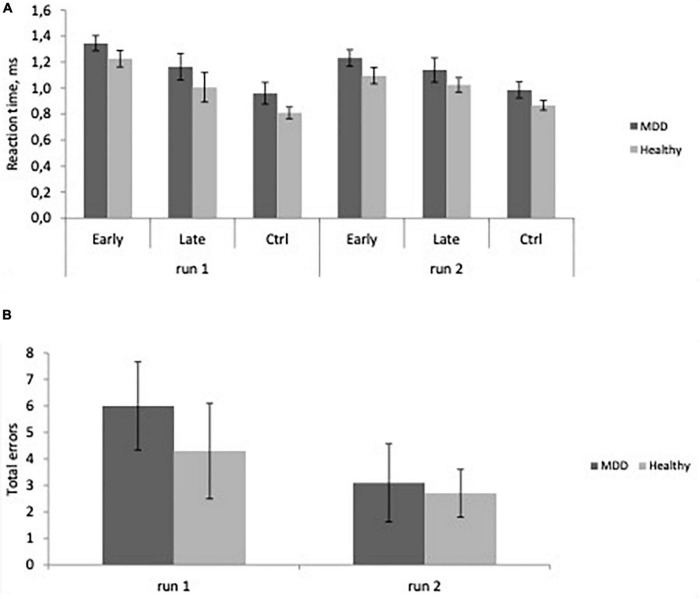
**(A)** Reaction time as a function of experience, Early learning, Late learning, Control (Ctrl) in major depressive disorder (MDD) patients, and Healthy participants in the first and second runs. Learning is evident as RT decreases from Early to Late trials (Mean ± SD). **(B)** Total errors across groups (Mean ± SD) in MDD and Healthy groups in the first and second runs.

Repeated measures analysis of variance showed a significant main effect of experience (*F* = 80.167, *p* < 0.0001), reflecting slower reaction time in early trials compared to late trials and control trials for all participants. There was no significant group difference (Figure 2A), no effect of age nor any significant interactions. There were no significant effects of group or age on error rate (Figure 2B).

### Imaging data

#### Brain activation during stimulus and feedback presentation

We examined brain activation differences between healthy and depressed patients and changes related to learning. Several brain regions exhibited the typical decline in activation with learning that we have observed previously. As with our previous research (Eliassen et al., 2003, 2012), these regions included bilateral medial superior frontal gyrus (BA6, 8), bilateral middle frontal gyrus (BA 9), and bilateral inferior frontal gyrus (anterior insula/frontal operculum; BA 44, 13) as well as parietal, temporal, and frontal polar regions.

**FIGURE 3:**
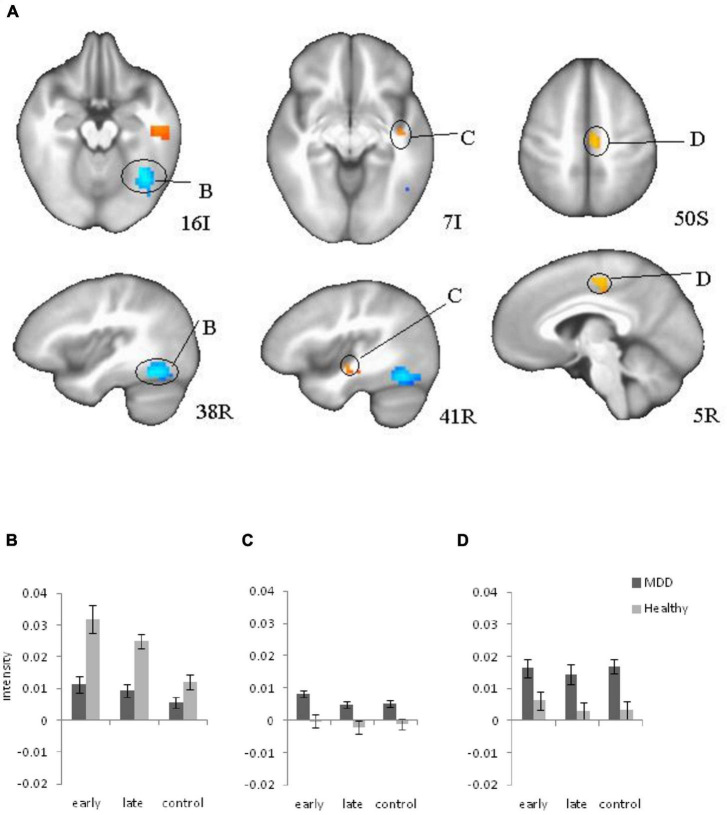
Brain regions that showed significant group differences between major depressive disorder (MDD) and Healthy for stimulus presentation during learning **(A)**, (B, C, D) Plots for ROI that displayed a significant difference between blood oxygen level dependent (BOLD) intensity in two groups. **(B)** Right Fusiform Gyrus; **(C)** Right insula; **(D)** Right Cingulate (motor area) cortex. Red color: MDD > Healthy; blue color: MDD < Healthy.

Regions that showed a significant difference between groups included right fusiform gyrus (BA 38), right insula (BA 21), and right cingulate motor area (BA 6) (Figure 3) for the stimulus presentation. Interestingly activation for stimulus presentation was higher in occipital cortex in healthy participants, but in depressed patients was higher in insula and cingulate cortex. *Post-hoc* analyses of these three active clusters indicated no significant modulation by age.

#### Atypical and melancholic scores and brain activation during learning

**FIGURE 4:**
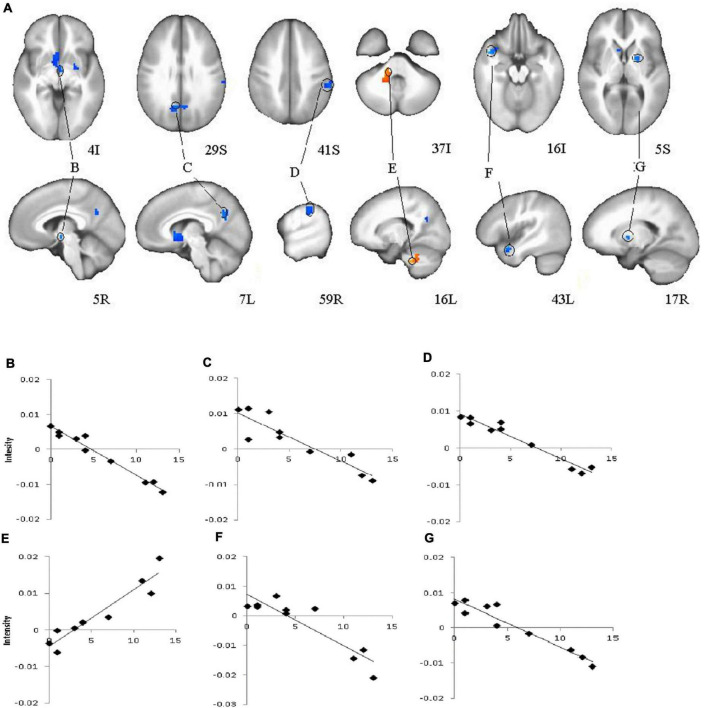
Brain regions that showed significant correlations for feedback presentation during learning and Atypical score in major depressive disorder (MDD) group **(A)**, (B, C, D, E, F, G) Plots for ROI that displayed a significant correlation between blood oxygen level dependent (BOLD) intensity and Atypical score. **(B)**–Right Thalamus, (including right hypothalamus, and left ventral caudate); **(C)**–Bilateral Precuneus (BA31); **(D)**–Right Inferior Parietal Lobule; **(E)**–Left Cerebellum; **(F)**–Left Superior Temporal Gyrus; **(G)**–Right Putamen. Red color–positive correlation; blue color–negative correlation.

Analyses of subtype scores revealed several brain areas where activation levels during feedback presentation were correlated with atypical score (Figure 4). BOLD signal intensity on feedback during learning (early and late trials) negatively correlated with atypical score in right thalamus (including right hypothalamus and left ventral caudate) bilateral precuneus (BA31) right putamen, right inferior parietal, and left superior temporal gyrus. Activation in left cerebellum showed the opposite pattern with activation increasing with higher atypical score. Melancholic scores correlated positively with activation during late feedback in bilateral precuneus and right caudate (Table 2). There is no significant correlation for early feedback or for combined early and late feedback.

**TABLE 2:** Regions with significant correlations between Melancholic score and feedback.

Cluster #	Cluster size (# voxels)	Center of mass			Peak activation
		*X*	*Y*	*Z*	
			
		(Local peak activation)			
1	52	−10.5	61.5	32.5	Right precuneus, Right BA7
2	46	13.5	79.5	23.5	Left cuneus, BA18
3	45	37.5	52.5	41.5	Left inferior parietal lobule, BA 40
4	40	−10.5	−4.5	2.5	Right caudate

## Discussion

The goal of the study was to identify differences between healthy individuals and MDD patients during reinforcement learning. Our results showed a similar learning effect in the behavioral and fMRI data: slower reaction time corresponded to increased BOLD intensity in the early learning trials compared to the late learning trials for both groups. The previous study (Eliassen et al., 2012) reported a similar learning effect in the behavioral and fMRI data in healthy participants. The current study observed the same effects in patients with MDD as well. The current study revealed significant differences in signals between healthy and depressed participants during learning. Correlations of the depression subtype scores to the BOLD signal are new findings of the brain activation changes during learning due to depression symptoms. Both groups showed decreased activation with learning, a pattern typical of healthy subjects according to our previous research (Eliassen et al., 2003, 2012). In comparison to healthy individuals, depressed patients showed elevated right insula and cingulate activation on stimulus presentation suggesting limbic hyperactivity during learning consistent with previous findings (Beauregard et al., 2006; Zhang et al., 2013). Elevated cingulate and insula activation may relate to general increased limbic activity in depression since it has been observed in the control trials as well. However, fMRI analyses revealed significant differences between groups in specific ROIs during the task performance and deviations became larger during the task performance in comparison to the already abnormal baseline activity, especially for decreased BOLD signal intensity in the right fusiform gyrus, and increased intensity in the right insula. Therefore, performing the task may provoke abnormal activity in learning circuits.

Greicius et al. (2007) reported abnormally increased functional connectivity in subgenual cingulate in depressed patients. Increased insula activation has been reported the neuronal basis of depression (Pandya et al., 2012). Additionally, anatomical studies showed that the gray matter volume of the insular cortex and the ACC correlated with depression symptoms (Mayberg, 1997). Electrophysiological studies on brain lateralization consider that the right hemisphere is hyperactive and the left hemisphere is hypoactive in patients with depression (Davidson, 1998; Allen and Reznik, 2015; Bruder et al., 2017). Our previous research confirmed left hypoactivity during the subsequent decision-making task in patients (Kustubayeva et al., 2020). FMRI study revealed changes in the right hemisphere in both directions depending on the brain structure.

We did not find significant reductions in activation on feedback presentation in MDD patients compared to healthy participants as shown in previous studies of reward hyposensitivity. Nevertheless, we must bear in mind that feedback during the current learning paradigm included negative feedback in early learning trials and positive feedback in the late learning that was predictable due to the easy task.

At the same time, in contrast to limbic regions, visual cortex activation was reduced in MDD patients compared to the control group, suggesting a decreased visual response to stimulus presentation (Schaefer et al., 2006; Knutson et al., 2008; Forbes et al., 2009; Smoski et al., 2009, 2011). This overall pattern suggests that limbic regions might activated to compensate for reduced visual cortical involvement, or that limbic overactivation disrupts visual cortical engagement. In either case, the limbic and cortical circuits are dysregulated in patients with MDD, which supports the characterization of MDD as a “multidimensional, system-level disorder” related to limbic-cortical circuit dysfunction (Mayberg, 1997, 2003).

A secondary aim of this study was to identify brain areas associated with atypical or melancholic features. The negative correlation with atypical score in thalamus, hypothalamus, and basal ganglia provides evidence of reward hyposensitivity in individuals with higher atypical scores. Higher melancholic scores were associated with higher brain activation in reward areas during late feedback (positive feedback only).

Interestingly, Foti et al. (2014) observed that event-related potential feedback negativity and fMRI ventral striatal responses to reward during guessing did not track melancholic or atypical features diagnosed by using the SCID. Authors found that feedback negativity (FN) was blunted in MDD subgroup with impaired mood reactivity. Therefore, further examination of the neural signature of depression subtypes and its diagnostic measurements is warranted to clarify these discrepant observations.

In conclusion, we observed differences in brain activation between MDD patients and healthy participants that provide evidence of limbic-cortical dysregulation during reinforcement learning that would not be predicted by reward hyposensitivity. On the other hand, brain activation in response to feedback correlated negatively with atypical features which suggests the presence of reward hyposensitivity in this subtype, and melancholic features revealed the opposite pattern. The presence of MDD is associated with dysregulation of neural circuits related to learning and subtype symptoms may alter the level of activity in some of these circuits.

## Limitations

This study had limitations that bear comment. Due to the study requirement to include only unmedicated clinical samples only 10 patients were included to this study. We included age as covariate in the analysis because of age differences with the ten healthy volunteers. Our sample comprised of 10 MDD individuals with a range of subtype features, so we chose to focus on variation in activation across the continuum of atypical and melancholic features. Also, because our task was limited to two runs and learning occurs on the first trial regardless of outcome, we cannot distinguish activation between positive or negative feedback in early learning trials.

## Data availability statement

Restrictions apply to the datasets. The datasets presented in this article are not readily available because our IRB protocol prohibits the public release of individual datasets. Requests to access the dataset should be directed to JE at james.eliassen@gmail.com.

## Ethics statement

The studies involving human participants were reviewed and approved by IRB, University of Cincinnati. The patients/participants provided their written informed consent to participate in this study.

## Author contributions

AK was involved in behavioral, fMRI data analysis, and writing of the manuscript. MS was involved in data analysis and writing the method section. JA was involved in task design, data acquisition, and revision of the manuscript. JE and EN contributed to the research idea, in providing the research finding, designing and conducting the research, supervising the data acquisition and processing, and revision of the manuscript. All authors contributed to the article and approved the submitted version.

## Footnotes

1
https://www.pstnet.com/

2
http://www.exelisvis.com/

3
https://www.loni.usc.edu
