# Corrigendum: Functional MRI study of feedback-based reinforcement learning in depression

**DOI:** 10.3389/fninf.2023.1175847

**Published:** 2023-03-21

**Authors:** Almira M. Kustubayeva, Erik B. Nelson, Michael L. Smith, Jane B. Allendorfer, James C. Eliassen

**Affiliations:** ^1^Center for Cognitive Neuroscience, Department of Physiology, Biophysics, and Neuroscience, Al-Farabi Kazakh National University, Almaty, Kazakhstan; ^2^Department of Psychiatry and Behavioral Neuroscience, College of Medicine, University of Cincinnati, Cincinnati, OH, United States; ^3^Department of Speech-Language-Hearing Sciences, University of Minnesota, Minneapolis, MN, United States; ^4^Department of Neurology, University of Alabama at Birmingham, Birmingham, AL, United States; ^5^Robert Bosch Automotive Steering LLC, Florence, KY, United States

**Keywords:** associative learning, reinforcement, functional magnetic resonance imaging (fMRI), major depressive disorder, brain activity

In the published article, there was a mistake in the **Funding** statement.

Original text: This research was supported by the National Alliance for Research on Schizophrenia & Depressio (NARSAD) Young Investigator Award and K23 MH067705 to EN and K01 DA020485 to JE. AK was supported by grant Ministry of Education and Science of the RK AP08856595. JA was supported by R01-DA022221-02S2 (PI: DelBello).

Due to an oversight by the authors during proofing, the order of grant support was listed incorrectly, and some awkward wording was not corrected. As a condition of supporting the research publication costs, the Ministry of Education and Science in the Republic of Kazakhstan requires that it must be listed first in the funding statement. The correct Funding statement appears below.

